# The Influence of Medical Culture and Key Factors on Mobile App Usage Frequency and Perceived Effectiveness in Physical Therapy: Cross-Sectional Study

**DOI:** 10.2196/68406

**Published:** 2025-06-12

**Authors:** Khalid A Alahmari, Ravi Shankar Reddy

**Affiliations:** 1 Program of Physical Therapy, Department of Medical Rehabilitation Sciences College of Applied Medical Sciences King Khalid University Abha Saudi Arabia

**Keywords:** app usage, medical culture, mobile health applications, perceived effectiveness, physical therapy

## Abstract

**Background:**

Medical culture refers to institutional attitudes toward technology usage, innovation, teamwork, and evidence-based practices. Hospitals with strong innovation cultures promote mobile app use, while traditional settings may resist change, relying on conventional methods. Medical culture significantly influences the usage of technology in health care, including mobile health (mHealth) applications, which have the potential to enhance patient care in physical therapy settings. Understanding the role of medical culture and other factors such as app usability and workplace setting in shaping the usage and perceived effectiveness of mobile apps is essential for promoting their integration into clinical practice.

**Objective:**

The study aimed to assess how medical culture influences health care professionals’ usage of mobile apps in clinical settings, particularly in physical therapy interventions, and to examine the relationship between the perceived effectiveness of mobile apps and cultural attitudes toward technology in health care teams.

**Methods:**

This cross-sectional study was conducted between April 2023 and March 2024 at a tertiary care hospital specializing in rehabilitation and physical therapy. A total of 456 health care professionals, including physical therapists and rehabilitation specialists, were surveyed. Medical culture was measured using a composite scale, while mobile app usage and perceived effectiveness were assessed through structured questionnaires.

**Results:**

Medical culture was strongly associated with mobile app usage frequency (*r*=0.54, *P*=.01), and multiple linear regression analysis showed that medical culture significantly predicted app usage (β=.45, *P*=.002). App usability, in terms of ease of use (β=.37, *P*=.008) and patient engagement features (β=.28, *P*=.02), also contributed to the perceived effectiveness of mobile apps. Hospitals showed higher usage and perceived effectiveness than private practices and rehabilitation centers. Years of experience (β=.32, *P*=.01) and app usage frequency (β=.45, *P*=.01) were additional significant predictors.

**Conclusions:**

This study highlights the critical role of medical culture in promoting the usage and perceived effectiveness of mobile apps in physical therapy. Positive cultural attitudes toward technology, combined with user-friendly app designs, can enhance the integration of mHealth tools in clinical settings. These findings underscore the importance of fostering an innovative medical culture and supporting the development of usable, patient-centered applications to optimize patient care.

## Introduction

Medical culture plays a fundamental role in shaping the attitudes, behaviors, and practices of health care professionals [1]. It encompasses the values, beliefs, and norms that govern how health care is delivered within an institution [2]. A positive medical culture, characterized by openness to innovation, collaboration, and evidence-based practice, can significantly influence the effectiveness and efficiency of health care delivery [3]. Conversely, a rigid or traditional medical culture can act as a barrier to the usage of new technologies and methods [4]. The role of medical culture extends beyond the attitudes of individual professionals, influencing how teams function and how institutions respond to emerging challenges in health care [5]. In recent years, there has been growing recognition of the importance of fostering a medical culture that encourages technological integration, teamwork, and continuous learning to improve patient care and outcomes [6].

As technology continues to transform health care, mobile apps have become essential tools for health care professionals, offering new ways to manage patient care, monitor health outcomes, and streamline clinical workflows [7]. However, the usage of these mobile apps is not uniform across clinical settings, with medical culture playing a pivotal role in determining the rate and success of their integration [8]. In clinical environments where innovation and technology are embraced, health care professionals are more likely to adopt mobile apps as part of their practice [9]. This is particularly relevant in physical therapy, where mobile apps can aid in exercise prescription, patient monitoring, and rehabilitation management. The usage of mobile health (mHealth) applications in physical therapy has the potential to enhance patient outcomes by providing real-time feedback, improving adherence to treatment plans, and increasing patient engagement [10]. Despite their benefits, the successful integration of these tools largely depends on the prevailing medical culture within the institution [10,11].

The perceived effectiveness of mHealth apps is another critical factor that influences their usage and sustained use by health care professionals [12]. Cultural attitudes toward technology, both at the individual and institutional levels, play a significant role in shaping perceptions of the value of mobile apps in clinical practice [13]. In physical therapy, where patient engagement and adherence to treatment are essential for successful outcomes, health care professionals are more likely to perceive mobile apps as effective tools if they work in environments that promote technological innovation and value patient-centered care [14]. A supportive medical culture that encourages the use of technology can enhance health care professionals’ perceptions of the utility and effectiveness of mobile apps, leading to more widespread usage [15]. However, in environments where there is resistance to change or skepticism about new technologies, health care professionals may be less likely to view mobile apps as beneficial, even if they have the potential to improve patient outcomes [16].

Despite the recognized benefits of mobile apps in clinical practice, there remains a significant gap in the literature regarding the role of medical culture in influencing their usage and perceived effectiveness, particularly in physical therapy [17]. While some studies have explored the broader factors that influence technology usage in health care, few have specifically examined the intersection of medical culture, mobile app usage, and perceived effectiveness in physical therapy settings [18]. In addition, most research has focused on larger health care systems or other clinical specialties, leaving a gap in understanding how mobile apps are adopted and used in physical therapy environments, where the dynamics of patient care and the nature of interventions may differ from other areas of health care [18]. Moreover, while usability factors such as ease of use and patient engagement features are known to influence the usage of technology, there is limited research on how these factors interact with medical culture to shape the perceived effectiveness of mobile apps [19].

The composite scale for assessing medical culture was adapted from the Organizational Culture Assessment Instrument (OCAI), which has been widely used in health care settings to evaluate openness to innovation, teamwork, and technology integration [20]. Mobile app usability was assessed using the System Usability Scale (SUS) [21], which evaluates ease of use, and the Patient Engagement Scale (PES) [22] to measure patient-centered app features. The perceived effectiveness of mobile apps was measured using the Mobile App Rating Scale (MARS), which assesses user experience and clinical impact [23].

This study seeks to address these gaps by examining the influence of medical culture on the usage of mobile apps in physical therapy and investigating the relationship between the perceived effectiveness of these applications and cultural attitudes toward technology. The study is particularly relevant given the growing importance of mHealth technologies in enhancing patient outcomes and improving clinical workflows in physical therapy. By focusing on the role of medical culture, this study aims to provide insights into how health care institutions can foster environments that promote the integration of mobile apps, thereby maximizing their potential to improve patient care [24]. Furthermore, understanding the factors that influence the perceived effectiveness of mobile apps can help health care professionals make informed decisions about their use and guide developers in designing tools that meet the needs of clinicians and patients alike [24].

The specific objectives of this study are 2-fold: first, to assess how medical culture influences health care professionals’ usage of mobile apps in clinical settings, with a focus on their use in physical therapy interventions, and second, to examine the relationship between the perceived effectiveness of mobile apps in physical therapy and the cultural attitudes toward technology in health care teams. We hypothesize that a positive medical culture, characterized by openness to innovation and collaboration, will be significantly associated with higher mobile app usage frequency rates and more favorable perceptions of app effectiveness in physical therapy. In addition, we hypothesize that usability factors such as ease of use and patient engagement features will moderate the relationship between medical culture and the perceived effectiveness of mobile apps.

## Methods

### Design and Setting

This observational cross-sectional study was conducted between April 2023 and March 2024 at the medical rehabilitation and physical therapy clinics affiliated with King Khalid University, Abha, Aseer Region, Kingdom of Saudi Arabia. Data were collected from health care professionals working in the physical therapy department, including therapists and rehabilitation specialists, using structured electronic surveys distributed through institutional email lists and departmental announcements.

### Participants

Participants in this study were health care professionals actively involved in patient care and the use of mHealth applications. Eligible participants had a minimum of 1 year of clinical experience in physical therapy and were either currently using or considering the use of mobile apps in their daily practice. Exclusion criteria included professionals not involved in direct patient care or those with limited exposure to mHealth technologies. A total of 612 invitations were sent, with 456 professionals ultimately participating. Participation was voluntary, with no incentives offered, and written informed consent was obtained before survey distribution. A convenience sampling method was used, selecting participants based on their active involvement in physical therapy and mHealth applications. Researchers accessed potential participants through institutional email lists and internal departmental communication channels, ensuring that invitations were sent directly to eligible professionals within the affiliated health care institutions.

### Variables

The primary variables of interest in this study were medical culture, mobile app usage frequency, and the perceived effectiveness of mobile apps. In this study, the primary outcomes were mobile app usage frequency, assessed by the frequency of app usage for patient care, and perceived effectiveness, measured using a structured Likert-scale questionnaire evaluating app impact on patient outcomes, engagement, and workflow efficiency. The independent variables included medical culture (measured using the OCAI), app usability (assessed via the SUS and PES), workplace setting (categorized as hospital, private practice, or rehabilitation center), and years of experience (self-reported by participants). Medical culture was assessed using a validated composite scale that measured the openness to innovation, teamwork, and the integration of technology within the health care setting [25]. Participants responded to a series of Likert-scale items ranging from 1 (strongly disagree) to 5 (strongly agree) [25]. The overall medical culture score was calculated as the mean of these items, with higher scores indicating a more positive medical culture [26]. The composite scale for assessing medical culture was based on previously validated tools used in health care settings (cite relevant studies or tools if applicable), and mobile app usability was assessed using a set of validated Likert-scale questions focusing on ease of use and patient engagement features [27].

Mobile app usage frequency was measured based on the frequency of mobile app usage, recorded as the number of days per week participants used mobile apps for patient care [28]. Participants were asked to indicate the types of mobile apps they used, including exercise apps, patient management tools, and monitoring apps [28]. The data on app usage frequency and types of apps used were self-reported via structured survey questions [28].

Perceived effectiveness of mobile apps was another key outcome, assessed using a Likert scale where participants rated the effectiveness of mobile apps in improving patient outcomes, enhancing patient engagement, and streamlining clinical workflows [29]. The effectiveness score was calculated as the mean of these ratings, with higher scores indicating greater perceived effectiveness [29].

Other variables included years of professional experience, categorized by the number of years working in physical therapy, and workplace settings, which were divided into hospitals, private practice, and rehabilitation centers [30]. Data on these variables were self-reported by participants in the survey [30]. In addition, app usability was evaluated through 2 key dimensions: ease of use and patient engagement features [31]. These dimensions were assessed via survey items where participants rated the usability of mobile apps in their clinical practice [31].

To ensure accuracy in the assessment of variables, all survey instruments used in this study were based on previously validated tools and tailored to the context of physical therapy. Data were collected through structured self-administered questionnaires distributed electronically, and responses were reviewed to ensure completeness and consistency before analysis.

### Sample Size Estimation

The sample size was determined using G*Power 3.1 (Heinrich Heine University Düsseldorf), which calculated a minimum requirement of 172 participants to detect a medium effect size (Cohen *f*²=0.15) with α=.05 and .80 statistical power for multiple regression with 5 predictors [32]. The medium effect size (Cohen *f*²=0.15) was selected based on findings from prior studies investigating technology usage in health care settings, where similar effect sizes have been reported in multiple regression analyses assessing factors influencing digital health integration [33]. A total of 612 invitations were sent, yielding 456 completed responses, surpassing the required sample size of 172 and ensuring sufficient statistical power for meaningful analysis.

### Data Analysis

Data analysis was conducted using SPSS (version 24; IBM Corp), and parametric statistical methods were used as the data followed a normal distribution, confirmed by the Shapiro–Wilk test. To address the first objective, multiple linear regression analysis was performed with mobile app usage frequency (number of days per week) as the dependent variable. Independent variables included medical culture score, years of experience, age, workplace setting, and interaction terms for medical culture × workplace setting. For the second objective, a separate multiple linear regression model was constructed with the perceived effectiveness of mobile apps as the outcome. Predictor variables included medical culture, app usability (ease of use and patient engagement features), app usage frequency, years of experience, and workplace setting. All variables were selected based on theoretical justification grounded in the existing literature on digital health usage and usability frameworks; no stepwise (forward or backward) procedures were applied. Interaction terms were included to examine the moderating effects of workplace settings on the association between medical culture and mobile app usage frequency. In addition, Pearson correlation was used to explore bivariate relationships among continuous variables, and ANOVA with Tukey post hoc tests compared mobile app usage and perceived effectiveness across different workplace settings and experience levels. A significance level of *P*<.05 was applied for all analyses.

### Ethical Considerations

This study was approved by the institutional review board of the hospital (REC#764-2023) on March 23, 2023, in accordance with the Declaration of Helsinki. Written informed consent was obtained from all participants before survey distribution and participation was voluntary, with the ability to opt-out at any stage. All collected data were anonymized to maintain confidentiality and prevent identification of individual participants. No financial or other compensation was provided to participants.

## Results

The study sample had an average age of 36.45 years with a majority of participants being male (62%) and having an average of 10.34 years of experience (Table 1). Participants were primarily used in hospitals (42%), followed by private practices (38%) and rehabilitation centers (20%). Mobile app usage was prevalent, with 70% of participants using mobile apps, most frequently exercise apps (65%). The mean medical culture score was relatively high (4.21 out of 5), while the perceived effectiveness of mobile apps was also favorable (3.87 out of 5). The majority of participants held either a master’s (50%) or a bachelor’s degree (40%), with a smaller proportion having a doctorate (10%).

**Table 1:** Demographic and clinical characteristics.

Variables		Values
Total participants, N (%)		456 (100)
Age (years), mean (SD), range		36.45 (8.52), 24-58
**Sex, n (%)**		
	Male	282 (62)
	Female	174 (38)
Years of experience, mean (SD), range		10.34 (5.23), 1-25
**Workplace setting, n (%)**		
	Hospital	191 (42)
	Private practice	173 (38)
	Rehabilitation Center	92 (20)
	Mobile app usage (days per week), mean (SD), range	3.45 (1.12), 1-7
**Type of mobile apps used, n (%)**		
	Exercise apps	296 (65)
	Patient management apps	182 (40)
	Monitoring apps	160 (35)
Medical culture score (out of 5), mean (SD), range		4.21 (0.67), 2.5-5
Perceived effectiveness (out of 5), mean (SD), range		3.87 (0.74), 2.8-5
**Education level, n (%)**		
	Bachelor’s degree	182 (40)
	Master’s degree	228 (50)
	Doctorate	46 (10)
**Use of mobile app, n (%)**		
	Yes	319, (70)
	No	137 (30)

**Figure 1:**
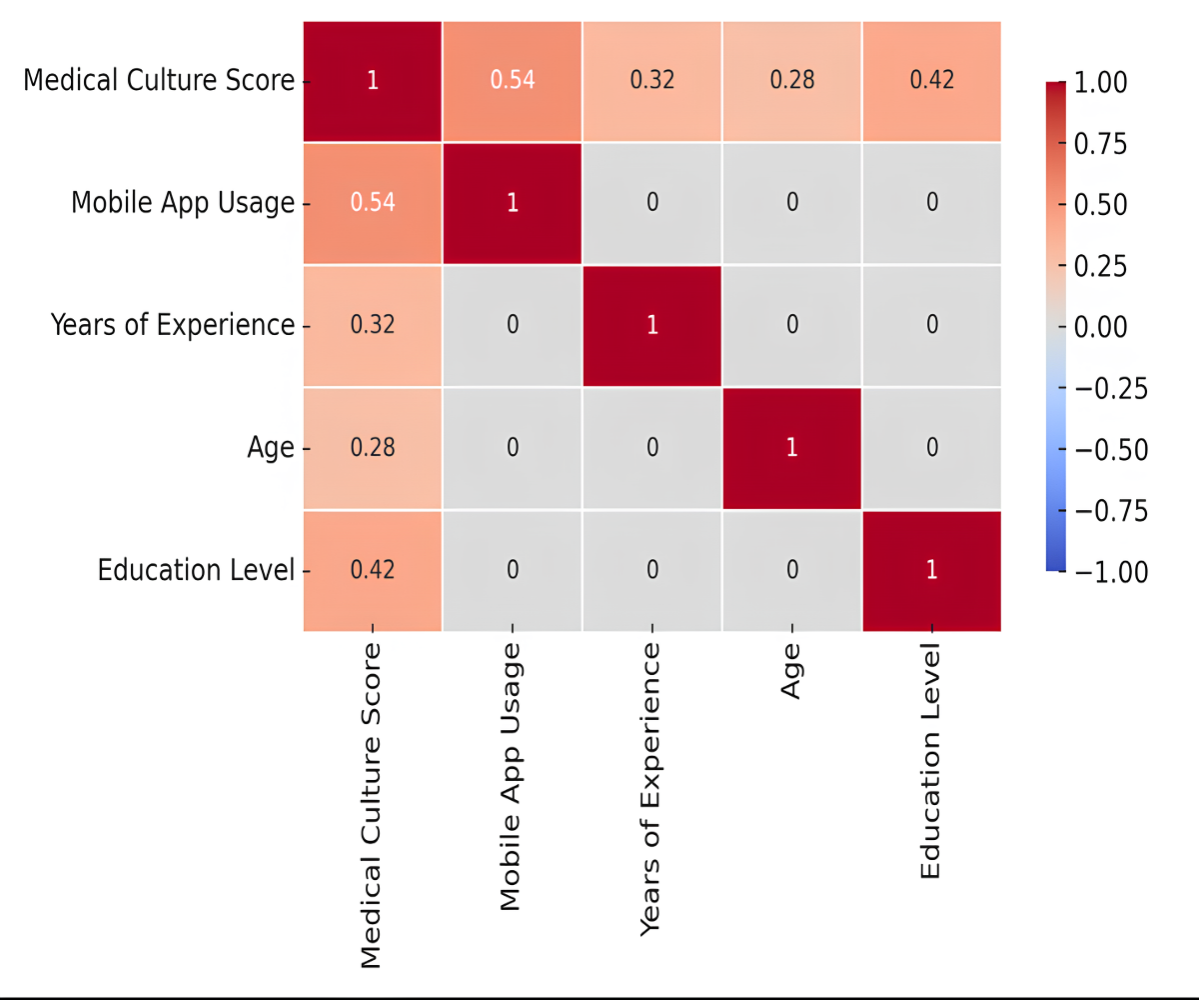
Pearson correlation matrix: influence of medical culture on mobile app usage, experience, age, and education.

The correlation analysis showed a significant positive relationship between medical culture and mobile app usage (*r*=0.54, *P*=.01), indicating that stronger medical culture promotes higher usage (Table 2). Years of experience (*r*=0.32, *P*=.04) and education level (*r*=0.42, *P*=.02) were also positively correlated with app usage, suggesting that experienced and highly educated professionals are more likely to use mobile apps. Age had a weaker, marginally significant correlation (*r*=0.28, *P*=.05). These relationships are summarized in Figure 1.

**Table 2:** Pearson correlation and influence of medical culture on mobile app usage frequency.

Variable	*r* (correlation coefficient)	*P* value
Medical culture score	1.0	—^a^
Mobile app usage (days per week)	0.54	.01
Years of experience	0.32	.04
Age (years)	0.28	.05
Education level	0.42	.02

^a^Not applicable.

Multiple linear regression analysis confirmed that medical culture significantly predicted mobile app usage frequency (β=.45, *P*=.002), with stronger medical culture linked to higher usage (Table 3). Years of experience (β=.32, *P*=.01) and workplace setting (hospital: β=.22, private practice: β=.12, rehabilitation center: β=.18) were also significant predictors. The relationship between medical culture and app usage was strongest in hospitals (interaction β=.28, *P*=.005) compared to private practices (β=.15, *P*=.04). The model demonstrated a good fit (*R*²=0.48, adjusted *R*²=0.46).

**Table 3:** Multiple linear regression analysis for mobile app usage frequency.

Predictor variable	β (coefficient)	SE	95% CI	*P* value	*R*²^a^	Adjusted *R*²^b^
Medical culture score	.45	0.07	0.31-0.59	.002	—^c^	—
Years of experience	.32	0.06	0.20-0.44	.01	0.48	0.46
Age (years)	.15	0.05	0.05-0.25	.03	—	—
Workplace setting (hospital)	.22	0.04	0.14-0.30	.02	—	—
Workplace setting (private practice)	.12	0.06	0.01-0.23	.04	—	—
Workplace setting (rehabilitation center)	.18	0.05	0.08-0.28	.03	—	—
Interaction: medical culture and hospital setting	0.28	0.08	0.12-0.44	.005	—	—
Interaction: medical culture and private practice	0.15	0.07	0.01-0.29	.04	—	—

^a^*R*²: coefficient of determination.

^b^Adjusted *R*²: adjusted coefficient of determination.

^c^Not applicable.

**Figure 2:**
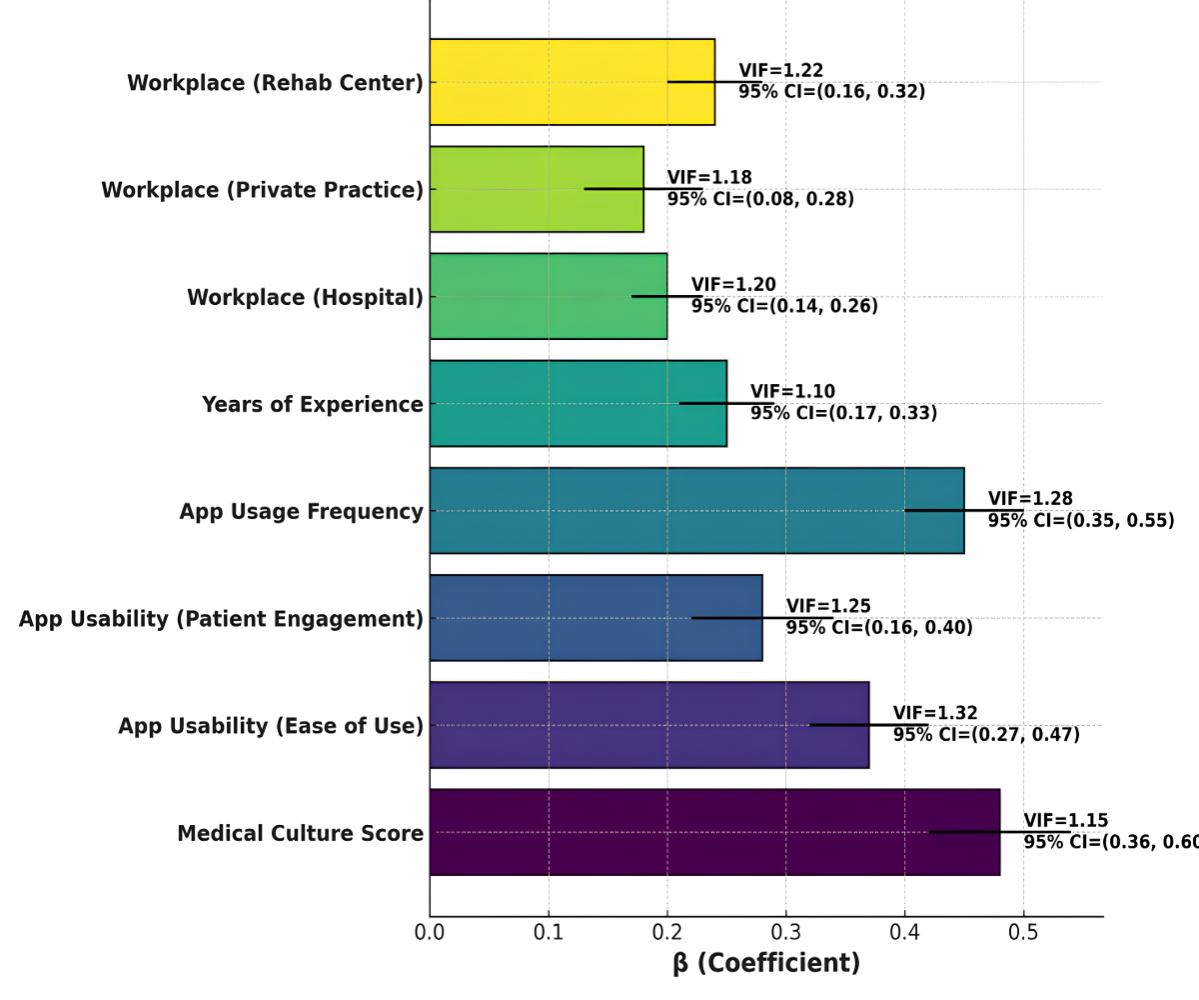
Abstract bar plot: medical culture and perceived effectiveness with variance inflation factor and CIs. VIF: variance inflation factor.

Medical culture strongly predicted the perceived effectiveness of mobile apps (β=.48, *P*=.001), enhancing their perceived value. Usability factors, including ease of use (β=.37, *P*=.008) and patient engagement features (β=.28, *P*=.02), also contributed positively (Table 4 and Figure 2). App usage frequency (β=.45, *P*=.01) and years of experience (β=.25, *P*=.02) were significant predictors. Perceived effectiveness was higher in hospitals (β=.20, *P*=.04) and rehabilitation centers (β=.24, *P*=.02). Low multicollinearity was confirmed (variance inflation factor < 1.5).

**Figure 3:**
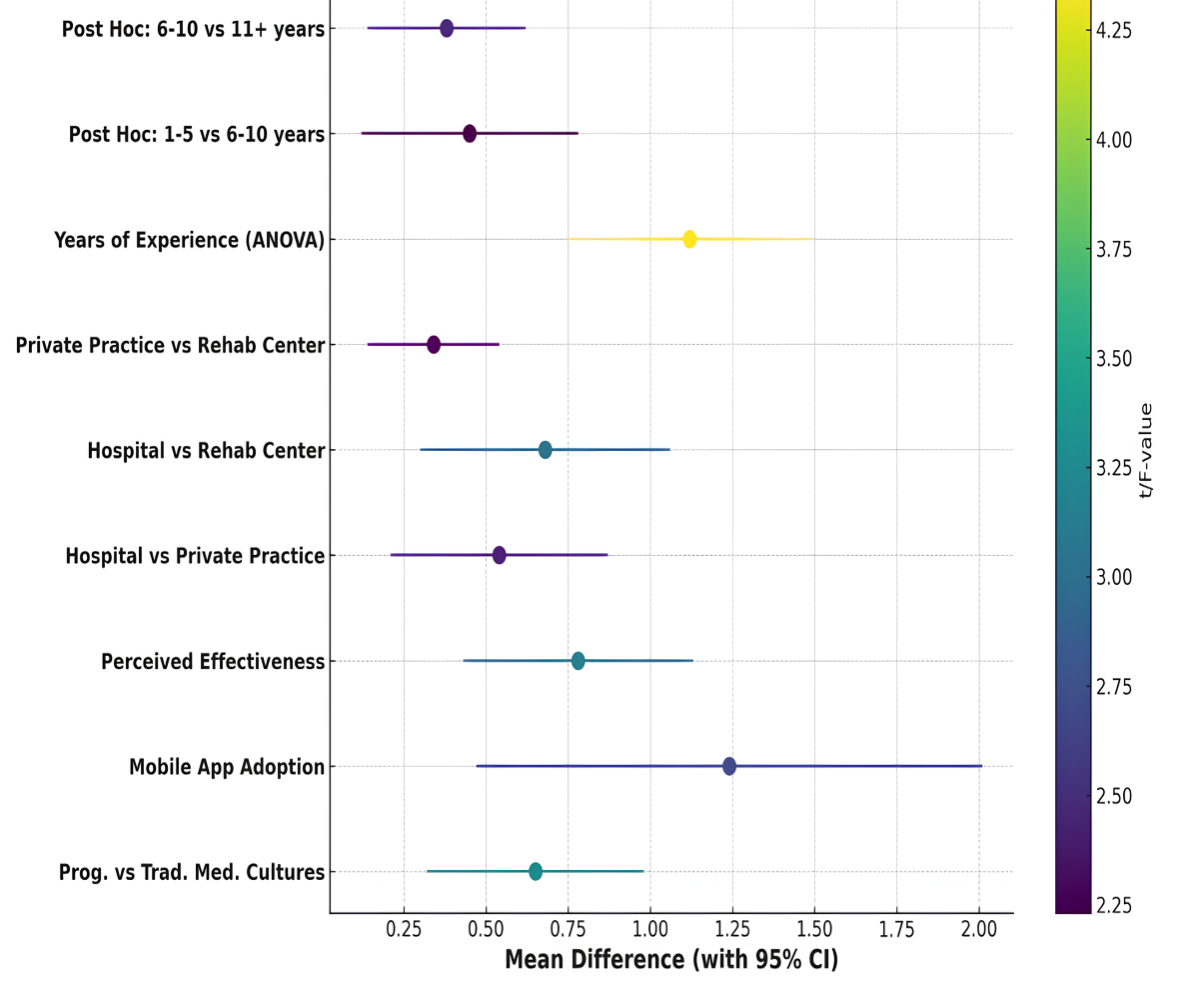
Subgroup analysis for mobile app usage frequency and perceived effectiveness (with workplace setting comparisons).

Subgroup analysis revealed that progressive medical cultures had significantly higher mobile app usage frequency than traditional cultures (mean difference=0.65, *P*=.002) and overall usage was greater (mean difference=1.24, *P*=.007; Table 5 and Figure 3). Perceived effectiveness was also higher (mean difference=0.78, *P*=.004). Usage and effectiveness were greater in hospitals compared to private practices (mean difference=0.54, *P*=.02) and rehabilitation centers (mean difference=0.68, *P*=.01), while private practices had higher usage than rehabilitation centers (mean difference=0.34, *P*=.03). Years of experience significantly influenced usage (*P*=.001), with post hoc analysis showing differences between 1-5 and 6-10 years (*P*=.03) and between 6-10 and 11+ years (*P*=.02).

**Table 4:** Relationship between medical culture and perceived effectiveness of mobile apps.

Variable	β (coefficient)	SE	95% CI	*P* value	VIF^a^
Medical culture score	0.48	0.06	0.36-0.60	.001	1.15
App usability (ease of use)	0.37	0.05	0.27-0.47	.008	1.32
App usability (patient engagement features)	0.28	0.06	0.16-0.40	.02	1.25
App usage frequency	0.45	0.05	0.35-0.55	.01	1.28
Years of experience	0.25	0.04	0.17-0.33	.02	1.1
Workplace setting (hospital)	0.2	0.03	0.14-0.26	.04	1.2
Workplace setting (private practice)	0.18	0.05	0.08-0.28	.03	1.18
Workplace setting (rehabilitation center)	0.24	0.04	0.16-0.32	.02	1.22

^a^VIF: variance inflation factor.

**Table 5:** Subgroup analysis for mobile app usage frequency and perceived effectiveness (with workplace setting comparisons).

Group comparison	Mean difference (95% CI)	Values	*P* value
Progressive vs traditional medical cultures	0.65 (0.32-0.98)	3.24 (454)^a^	.002
Mobile app usage frequency	1.24 (0.47-2.01)	2.71 (454)^a^	.007
Perceived effectiveness	0.78 (0.43-1.13)	3.12 (454)^a^	.004
Workplace setting: hospital vs private practice	0.54 (0.21-0.87)	2.45 (362)^a^	.02
Workplace setting: hospital vs rehabilitation center	0.68 (0.30-1.06)	3.01 (281)^a^	.013
Workplace setting: private practice vs rehabilitation center	0.34 (0.14-0.54)	2.31 (263)^a^	.03
Years of experience (ANOVA)	1.12 (0.75-1.49)	4.35 (2, 453)^b^	.001
Tukey post hoc: 1-5 years vs 6-10 years	0.45 (0.12-0.78)	2.23 (453)^a^	.03
Tukey post hoc: 6-10 years vs 11+ years	0.38 (0.14-0.62)	2.49 (453)^a^	.02

^a^*t* test (*df*) value.

^b^*F* test (*df*) value.

## Discussion

### Principal Results

This study aimed to assess how medical culture influences mobile app usage frequency in clinical settings and to examine the relationship between the perceived effectiveness of mobile apps and various demographic and workplace factors. The results demonstrate that a strong medical culture is significantly associated with higher mobile app usage frequency, as indicated by both the correlation and regression analyses. In addition, key factors such as years of experience, workplace setting, and app usability (ease of use and patient engagement features) significantly contributed to both app usage and perceived effectiveness. Subgroup analysis further revealed that progressive medical cultures promoted greater mobile app usage frequency compared to traditional settings, with hospitals showing the highest levels of usage and perceived effectiveness. The positive impact of workplace settings, particularly hospitals, was also observed in comparison to private practices and rehabilitation centers, with significant differences in app usage patterns across experience levels.

### Comparison With Prior Work

The findings from this study suggest that medical culture plays a crucial role in influencing mobile app usage frequency in clinical settings. The positive correlation between medical culture and app usage (*r*=0.54) indicates that health care environments that promote innovation, collaboration, and openness to technology are more likely to integrate mHealth applications into their routine practices [34]. The significant role of years of experience (*r*=0.32) and education level (*r*=0.42) further suggests that more experienced and highly educated health care professionals are likely to be early adopters of mHealth technologies, possibly due to greater familiarity with evidence-based practices and a stronger inclination to use tools that enhance patient care [35]. Age, while marginally significant (*r*=0.28), showed a weaker relationship with app usage, which could be due to generational differences in comfort with technology or the perceived need for such tools among older professionals [36]. The regression analysis reinforced these findings, demonstrating that medical culture, workplace setting, and years of experience significantly predict mobile app usage, particularly in hospitals where app usage was found to be stronger than in private practices or rehabilitation centers [37]. These results highlight the importance of fostering supportive institutional cultures that encourage the integration of digital tools in health care [38].

Beyond resistance to change, additional barriers to mobile app usage frequency include data privacy concerns, regulatory challenges, and technical support limitations. Health care professionals may be hesitant to adopt mobile apps due to concerns regarding patient confidentiality and adherence to institutional security protocols [15]. Regulatory uncertainty surrounding digital health tools can also create hesitancy, as compliance requirements vary across institutions and regions [39]. Furthermore, limited technical support and inadequate training resources may impede successful implementation, particularly in smaller health care settings [40]. Addressing these barriers through enhanced data security measures, clear regulatory guidance, and improved training programs may facilitate broader usage of mobile apps in clinical practice.

Hospitals are more likely to adopt mobile apps due to several key factors. They typically have greater financial resources and institutional support for implementing new technologies, along with dedicated IT infrastructure and technical assistance [41]. In addition, hospitals often follow standardized policies for digital health integration, which facilitates widespread usage [41]. In contrast, private practices may face financial limitations, have less access to technical support, and rely on individual practitioners’ discretion for technology usage, leading to variability in usage [42]. Addressing these challenges by providing financial incentives, technical training, and policy guidance for private practices may help bridge the gap in mobile app usage frequency.

These results are consistent with previous studies that have identified the influence of organizational culture and professional experience on technology usage in health care [43]. A study by Kruszyńska-Fischbach et al [44] found that health care settings with a strong culture of innovation and teamwork were more likely to adopt eHealth technologies, aligning with this study’s findings on the role of medical culture [44]. Similarly, Alsyouf et al [45] highlighted the importance of workplace factors, showing that hospitals with supportive leadership and an openness to technological advancements tend to experience higher rates of health IT usage [45]. The significance of education and experience is also well documented; Almaiah et al [46] Diffusion of Innovations theory emphasizes that individuals with greater knowledge and expertise are more likely to adopt new technologies [46]. Thus, the results of this study are in line with existing literature, underscoring the combined influence of medical culture, professional experience, and workplace dynamics in shaping the use of mHealth applications in clinical practice [46].

The results of this study suggest that medical culture plays a pivotal role in shaping the perceived effectiveness of mobile apps in clinical practice. A positive and supportive medical culture significantly enhances the perceived value of these tools, as evidenced by the strong correlation between medical culture score and perceived effectiveness (β=.48) [46]. In addition, app usability, both in terms of ease of use (β=.37) and patient engagement features (β=.28), emerged as crucial factors that contributed to higher perceived effectiveness [47]. This highlights that mobile apps that are intuitive and promote active patient involvement are more likely to be perceived as effective by health care professionals [47]. The frequency of app usage (β=.45) and years of experience (β=.25) also played significant roles in determining app effectiveness, indicating that experienced professionals who use mobile apps more frequently are more likely to see their benefits [48]. Workplace setting further influenced perceived effectiveness, with hospitals (β=.20) and rehabilitation centers (β=.24) showing higher scores than private practices [49].

These findings align with existing literature, which consistently emphasizes the influence of workplace culture and usability on the effectiveness of health technologies [50]. Srisathan et al [51] demonstrated that organizational culture supporting innovation and technology integration significantly impacts the perceived benefits of health IT systems, a result mirrored in this study [51]. Similarly, Hajesmaeel-Gohari et al [52] found that ease of use and engagement are key usability factors that enhance the acceptance and effectiveness of health care applications [52]. Furthermore, studies by Heidt et al [53] and Witter et al [54] corroborate the importance of workplace settings, with hospitals often being more resource-rich and technologically advanced environments compared to private practices, thereby fostering higher mobile app usage frequency and perceived effectiveness [53]. These findings reinforce the critical role that institutional support and user-friendly design play in promoting the successful implementation and perceived value of mHealth applications in clinical practice [54].

The clinical significance of this study lies in its demonstration of how a positive medical culture, combined with workplace support and usability of mHealth applications, can significantly enhance the usage and perceived effectiveness of these tools in clinical practice [55]. By identifying key factors such as medical culture, years of experience, and app usability, this study provides critical insights into how health care institutions can foster environments that encourage the integration of mobile apps to improve patient care [56]. In particular, the findings underscore the importance of creating a culture of innovation and technological openness in health care settings, especially in hospitals and rehabilitation centers, where app usage and perceived effectiveness were found to be higher [57]. This study suggests that, with proper institutional support and user-friendly app design, mHealth applications can serve as effective tools for enhancing clinical workflows, improving patient engagement, and ultimately leading to better patient outcomes.

### Limitations

Despite its valuable insights, this study has several limitations. The cross-sectional design prevents establishing causality between medical culture and mobile app usage frequency, limiting conclusions about the direction of influence. Self-reported data may introduce biases such as social desirability or recall bias. While the sample was diverse, it was restricted to specific health care settings, affecting generalizability. In addition, the study focused on medical culture, experience, and app usability but did not explore factors like patient perspectives, cost-effectiveness, and technical infrastructure. Response bias is another potential limitation, as demographic data on nonrespondents were not collected. Although the response rate was high (74.5%), differences in experience, workplace setting, or openness to technology usage among nonrespondents cannot be ruled out. Future research should include nonresponder analysis to improve external validity and use longitudinal designs to better understand causal relationships. Furthermore, as the study was conducted in Saudi Arabia, cultural and regulatory differences may limit its applicability to other regions. The hierarchical nature of health care institutions and regulatory policies in Saudi Arabia may differ from decentralized or patient-driven models elsewhere. Variations in data privacy laws, reimbursement structures, and digital health policies could also impact mobile app usage frequency differently across health care systems. Future studies should examine these factors in diverse geographic and regulatory contexts while also assessing patient-related outcomes and the economic and technical feasibility of mHealth application integration.

### Conclusions

This study demonstrates that a strong medical culture, along with workplace support and app usability, are critical factors driving the usage and perceived effectiveness of mHealth applications in clinical settings. Health care professionals working in environments that promote innovation and technology integration, particularly in hospitals and rehabilitation centers, are more likely to adopt and perceive mobile apps as beneficial tools for patient care. In addition, professionals with more experience and those using apps frequently reported higher perceived effectiveness. The findings highlight the importance of fostering a culture of openness to technology, coupled with designing user-friendly apps that enhance patient engagement. These insights provide actionable recommendations for health care institutions seeking to optimize the integration of mHealth technologies to improve clinical workflows and patient outcomes.

## Abbreviations

OCAI: Organizational Culture Assessment Instrument
MARS: Mobile App Rating Scale
mHealth: mobile health
PES: Patient Engagement Scale
SUS: System Usability Scale
